# Rational Design of Ni(OH)_2_ Hollow Porous Architecture for High-Sensitivity Enzyme-Free Glucose Sensor

**DOI:** 10.1186/s11671-018-2726-8

**Published:** 2018-10-29

**Authors:** Liangliang Tian, Gege He, Meijing Chen, Jinbiao Wang, Yucen Yao, Xue Bai

**Affiliations:** 1 Research Institute for New Materials Technology, Chongqing University of Arts and Sciences, Chongqing, People’s Republic of China; 2 School of Science, MOE Key Laboratory for Non-equilibrium Synthesis and Modulation of Condensed Matter, State Key Laboratory for Mechanical Behavior of Materials, Xi’an Jiaotong University, Xi’an, People’s Republic of China

**Keywords:** Ni(OH)_2_, Hollow structure, Coordinating etching and precipitating, Glucose detection, Electrochemical sensor

## Abstract

Ni(OH)_2_ electrocatalysts have acquired lots of research attentions as ideal substitutes for noble metals. However, their electrocatalytic performance still cannot meet the demands for applications due to the difficulties in electron transfer and mass transport. According to kinetics principle, the construction of hollow structure is regarded as an effective method to achieve outstanding electrocatalytic performance. In this work, Ni(OH)_2_ hollow porous architecture (Ni(OH)_2_ HPA) was simply synthesized through a coordinating etching and precipitating (CEP) method for the building of enzymatic-free glucose sensors. Ni(OH)_2_ HPA presents large specific surface area (SSA), ordered diffusion channels, and structure stability. As a detection electrode for glucose, Ni(OH)_2_ HPA exhibits eminent electroactivity in terms of high sensitivity (1843 μA mM^−1^ cm^−2^), lower detection limit (0.23 μM), and short response time (1.4 s). The results demonstrate that Ni(OH)_2_ HPA has practical applications for construction of enzymatic-free electrochemical sensors. The design of hollow structure also provides an effective engineering method for high-performance sensors.

**Electronic supplementary material:**

The online version of this article (10.1186/s11671-018-2726-8) contains supplementary material, which is available to authorized users.

## Background

Glucose detection is very important in clinical biochemistry, food processing, and environmental monitoring. Developing a fast and reliable sensing method for glucose is an urgent demand for these applications [1–3]. Many techniques have been developed for this purpose, such as surface plasmon resonance [4], Fehling reagent method [5], optical rotation method [6], fluorescence [7], and electrochemistry [8]. Among these techniques, electrochemical methods have attracted more attentions owing to its high sensitivity, simplicity, low cost, and extraordinary low detection limit [9].

It is well known that the electrocatalytic activity of the working electrode determines the performance of the electrochemical sensors. Therefore, the design of electrode materials is vital for electrochemical sensors. Recently, transition metal hydroxides have been widely researched in this field due to the advantages of large reserves, low cost, and high activity derived from the redox of metal composition [10]. Typically, Ni(OH)_2_ was recognized as an ideal catalyst for glucose due to the redox couple (Ni^3+^/Ni^2+^) in alkaline medium. Although the metal components of Ni(OH)_2_ can be used to restore the highly active electrons provided by the redox, their catalytic activity is still not high enough to meet the large-scale industrial production and people’s living needs because of the difficulties in electron transfer and mass transport.

Inspired by the intimate connection between kinetics and microstructures (shape, size, component), scientists have already built different structured nanomaterials that are good for electrocatalytic dynamics, since the properties of the nanomaterials are usually structure-dependent [11]. Hollow porous nanostructure, which possesses well-defined interior voids, high specific surface area (SSA), low density, and structure stability, has attracted growing interests in recent years [12]. The available inner cavities effectively prevent active particles from aggregation and accommodate the structural strain accompanied with long-time measurements [13]. Otherwise, the functional shells can offer larger contact area between electrolyte and electrode, provide sufficient active sites, and reduce the length for both mass and electron transport [14]. Furthermore, the porous thin shell also supplies enough diffusion paths for analyte and intermediates, which are good for mass transport process [15]. In conclusion, high-active Ni(OH)_2_ electrocatalysts can be acquired through the building of hollow porous feature.

Herein, cubic Ni(OH)_2_ HPA is constructed by a Cu_2_O-templated method inspired by the concept of coordinating etching and precipitating (CEP) route [16]. In order to demonstrate the advantages of hollow porous architecture, we comparatively evaluated the electrocatalytic activity of Ni(OH)_2_ HPA and broken Ni(OH)_2_ HPA (Ni(OH)_2_ BHPA) through the detection of glucose. The hollow porous architecture provides larger SSA, more ordered transfer paths, and higher electron transfer efficiency compared to Ni(OH)_2_ BHPA. Thus, the as-prepared Ni(OH)_2_ HPA electrode presents higher electrocatalytic activity in terms of higher sensitivity, lower detection limit, and faster response time. The results demonstrate that Ni(OH)_2_ HPA has potential applications for construction of electrochemical glucose sensors. This facile strategy also provides a valid method in the development of highly efficient nanomaterials for electrochemical sensors.

## Methods/Experimental

### Chemicals and Reagents

Copper chloride (CuCl_2_·2H_2_O; ≥ 99.0%), nickel chloride (NiCl_2_·6H_2_O; ≥ 98.0%), sodium thiosulfate (Na_2_S_2_O_3_·5H_2_O; ≥ 99.0%), polyvinyl pyrrolidone (PVP; *M*_*w*_ = 40,000), and sodium hydroxide (NaOH; ≥ 98.0%) were obtained from Chengdu Kelong. Glucose (Glu.; ≥ 99.5%), lactose (Lact.; ≥ 98.0%), sucrose (Sucr.; ≥ 99.5%), fructose (Fruc.; ≥ 99.0%), l-ascorbic acid (AA; ≥ 99.7%), uric acid (UA; ≥ 99.0%), and Nafion solution (5 wt% in mixture of lower aliphatic alcohols and water) were obtained from Sigma-Aldrich.

### Synthesis of Ni(OH)_2_ HPA

Firstly, cubic Cu_2_O crystals were prepared following our previous work (Additional file 1: Figure S1) [17]. Then, 10 mg as-prepared cubic Cu_2_O crystals and NiCl_2_·6H_2_O powder (4 mg) were dispersed into a mixed ethanol-water solution (10 mL, volume ratio = 1:1) by ultrasound. Thereafter, 0.33 g PVP powder was added with vigorous stirring for 0.5 h. Then, Na_2_S_2_O_3_ (4 mL, 1 M) was added dropwise into the above system. The reaction was proceeded at normal temperature (25 °C) for 3 h. Finally, the products were washed for several times by centrifugation and dried at normal temperature. Ni(OH)_2_ BHPA was obtained as contrast sample through strong ultrasonic treatment of Ni(OH)_2_ HPA for 2 h (Additional file 1: Figure S2).

### Materials Characterization

The crystal structure and composition of the products were measured by X-ray diffraction (XRD; Rigaku D/Max-2400) and X-ray photoelectron spectrometer (XPS; ESCALAB250Xi). The morphologies of the products were characterized by field emission scanning electron microscope (FESEM; FEI Quanta 250 and Zeiss Gemini 500) and high-resolution transmission electron microscope (HRTEM; FEI F20). The SSA and pore structure were analyzed on Brunauer-Emmett-Teller (BET; Belsort-max).

### Electrochemical Measurements

All electrochemical measurements were operated on electrochemical workstation (μIII Autolab). The working electrode is prepared by casting Nafion-impregnated Ni(OH)_2_ HPA (or Ni(OH)_2_ BHPA) powders onto a glassy carbon electrode (GCE; 3 mm in diameter) at room temperature. Specifically, 5 μL of the suspension (1 mg/ml in 0.05% Nafion solution) is dropped onto the pretreated GCE and dried by flowing N_2_. A Pt foil and Ag/AgCl electrode were used as counter electrode and reference electrode, respectively. The electrocatalytic activity of working electrodes was measured by cyclic voltammetry (CV), chronoamperometry (CA), and electrochemical impedance spectroscopy (EIS). EIS data were collected between 0.01 and 100 kHz with a perturbation amplitude of 5 mV.

## Results and Discussions

### Characterizations

**Fig. 1:**
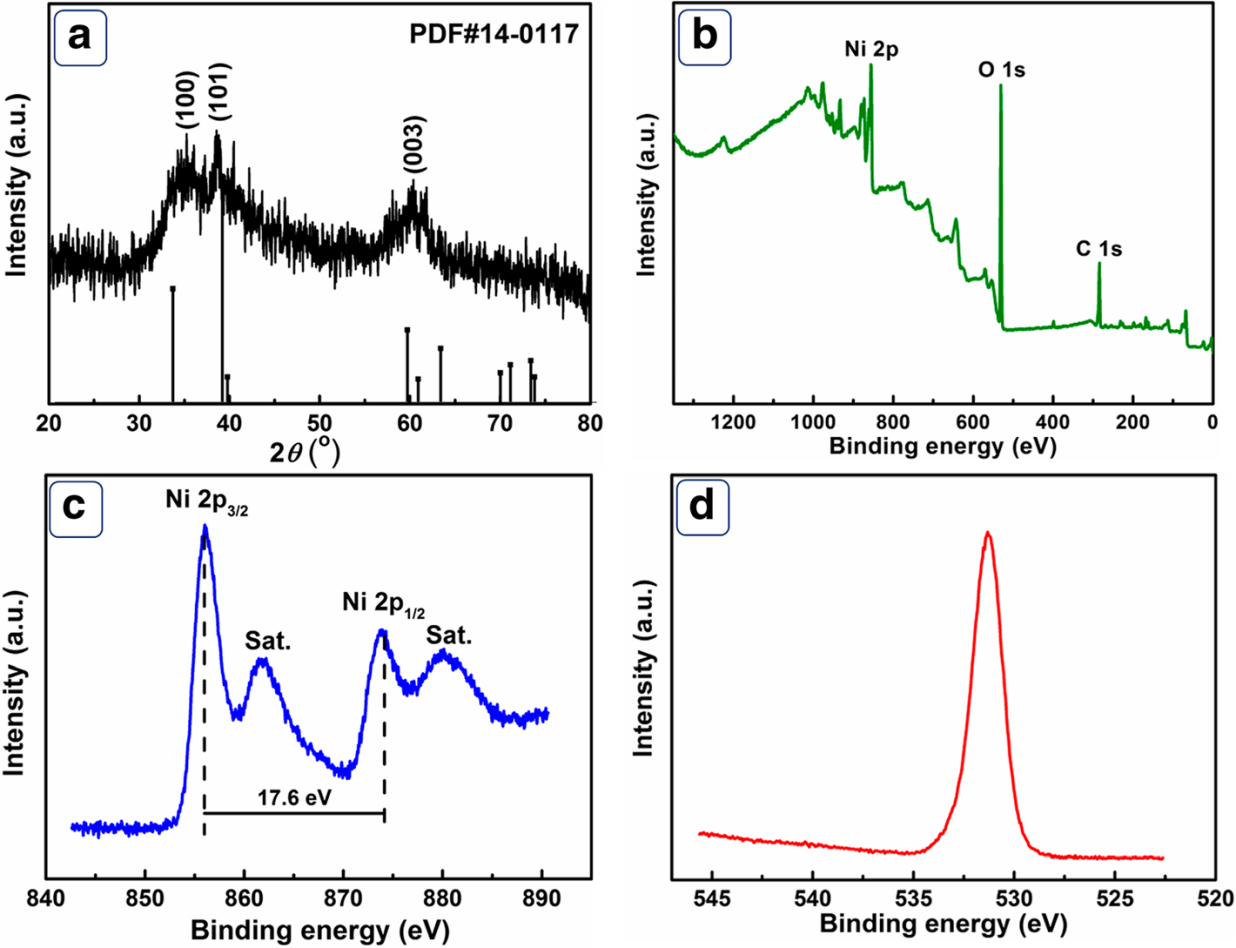
**a** The XRD pattern of prepared Ni(OH)_2_. The XPS spectra for the products. **b** Survey. **c** Ni 2p. **d** O 1s

The XRD pattern of Ni(OH)_2_ products was shown in Fig. 1a. The three main diffraction peaks can be assigned to (100), (101), and (003) crystalline planes of hexagonal *β*-Ni(OH)_2_ (JCPDS no. 14-0117) [18]. The weak intensity of the diffraction peaks can be attributed to low crystallinity of the products. The purity and composition of the as-prepared Ni(OH)_2_ were further investigated by XPS. The survey spectrum (Fig. 1b) demonstrates O 1s and Ni 2p peaks, revealing the main composition of the products. As displayed in Fig. 1c, the peaks located at 856.1 eV and 873.7 eV can be assigned to Ni 2p_3/2_ and Ni 2p_1/2_, respectively. A binding energy separation of 17.6 eV is clearly observed, which is the characteristic of *β*-Ni(OH)_2_. As displayed in Fig. 1d, the single peak at 531.2 eV corresponds to Ni–O–Ni bond in Ni–OH. By comparing the data with previous XPS studies, the presented Ni and O can be assigned to Ni^2+^ and OH^−^ in Ni(OH)_2_, respectively [16]. The analysis of XPS and XRD confirm the successful preparation of Ni(OH)_2_ phase.

**Fig. 2:**
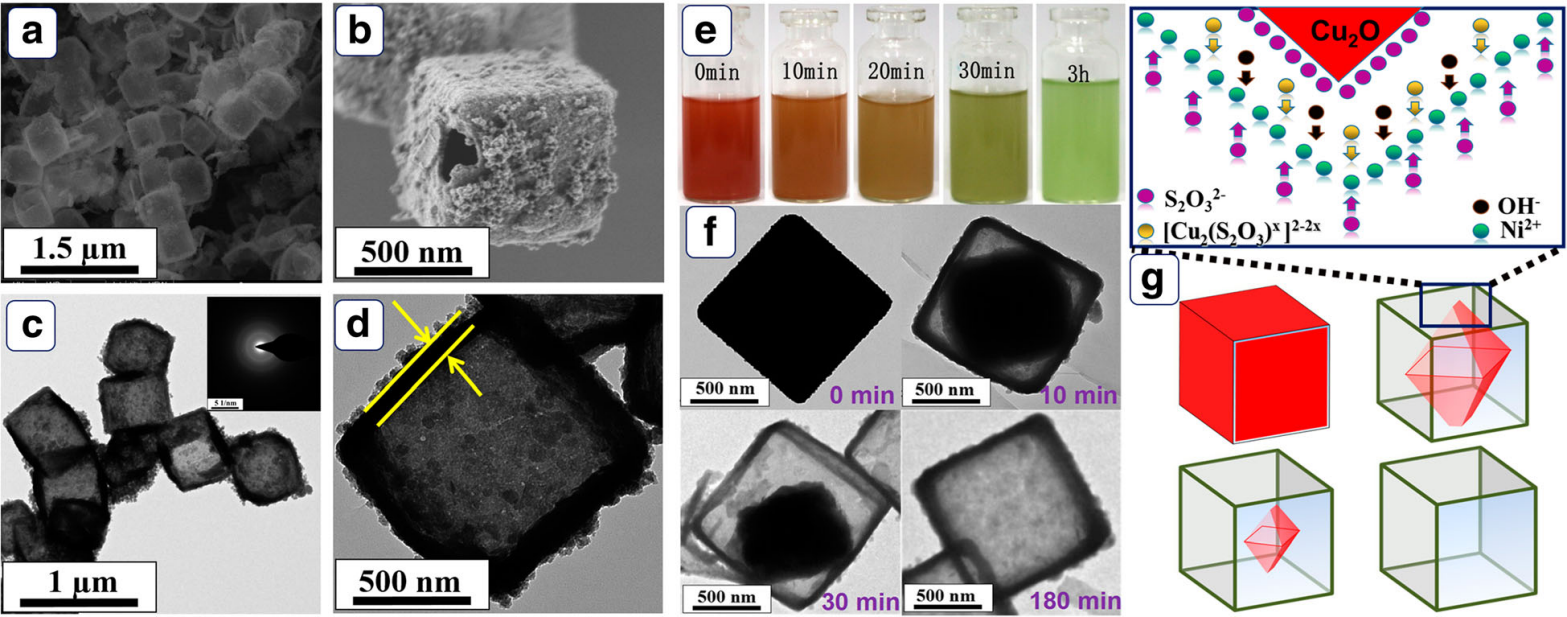
**a**, **b** SEM and **c**, **d** TEM images of the Ni(OH)_2_ HPA; inset of **c** is the SAED pattern of Ni(OH)_2_ HPA. **e** Optical photographs of the reaction solution at different time after addition of etchant. **f** TEM images of the products monitored at different reaction time. **g** Schematic illustration of the proposed growth mechanism of Ni(OH)_2_ HPA

The low-magnification SEM image in Fig. 2a demonstrates a uniform cubic feature of the prepared Ni(OH)_2_ products. The partly broken cube shown in Fig. 2b confirms the hollow characteristic of Ni(OH)_2_ HPA. Moreover, the shell of Ni(OH)_2_ HPA is built through the aggregation of numerous fine nanoparticles, making the shell rough and porous. The TEM images displayed in Fig. 2c further confirms the hollow structure of Ni(OH)_2_ products. Meanwhile, no significant diffraction ring is observed in selected area electron diffraction (SAED) pattern, suggesting low crystallinity of Ni(OH)_2_ HPA. This result agrees well with the observation of XRD. Clearly investigated in Fig. 2d, the Ni(OH)_2_ hollow cube has an edge length of ~ 600 nm and a shell thickness of ~ 50 nm. The hollow porous structure provides large SSA and amounts of diffusion channels, which may benefit the mass diffusion process, resulting in satisfactory electrocatalytic activity.

The products prepared at different reaction stages were centrifuged and observed to realize the relevant formation principle. As observed in Fig. 2e, the color of the reaction system gradually turns light green and precipitates generate at the same time. As shown in Fig. 2f, the interior Cu_2_O cores are gradually etched to octahedron after the addition of S_2_O_3_^2−^ ions. The Cu_2_O octahedrons finally disappear with the increase of reaction time. Combined with TEM images, the formation principle is illustrated in Fig. 2g. Apparently, S_2_O_3_^2−^ ions adsorbed around Cu_2_O cubes play versatile roles during the formation process of Ni(OH)_2_ HPA: (i) soluble [Cu_2_(S_2_O_3_^2−^)_x_]^2−2x^ complex is formed through the combination of Cu^+^ ions and S_2_O_3_^2−^ (reaction (1)) and simultaneously OH^−^ ions are released. (ii) The hydrolysis of S_2_O_3_^2−^ also releases OH^−^ ions (reaction (2)). (iii) Reactions (1) and (2) facilitate the formation of Ni(OH)_2_ (reaction (3)) [19]. Regarding the kinetics factors, the diffused OH^−^ ions from the interior determine the formation of Ni(OH)_2_ shell. Furthermore, the etching of Cu_2_O is correlated to the transport of S_2_O_3_^2−^ from exterior into internal space [20]. Synchronously controlling of OH^−^ and S_2_O_3_^2−^ transport leads to the formation of well-defined Ni(OH)_2_ HPA.

1 $$ {\mathrm{Cu}}_2\mathrm{O}+x{\mathrm{S}}_2{{\mathrm{O}}_3}^{2-}+{\mathrm{H}}_2\mathrm{O}\to {\left[{\mathrm{Cu}}_2{\left({\mathrm{S}}_2{\mathrm{O}}_3\right)}_x\right]}^{2-2x}+2{\mathrm{O}\mathrm{H}}^{-} $$

2 $$ {\mathrm{S}}_2{{\mathrm{O}}_3}^{2-}+{\mathrm{H}}_2\mathrm{O}\rightleftharpoons {\mathrm{H}\mathrm{S}}_2{{\mathrm{O}}_3}^{2-}+{\mathrm{O}\mathrm{H}}^{-} $$

3 $$ {\mathrm{Ni}}^{2+}+2{\mathrm{OH}}^{-}\to \mathrm{Ni}{\left(\mathrm{OH}\right)}_2 $$

**Fig. 3:**
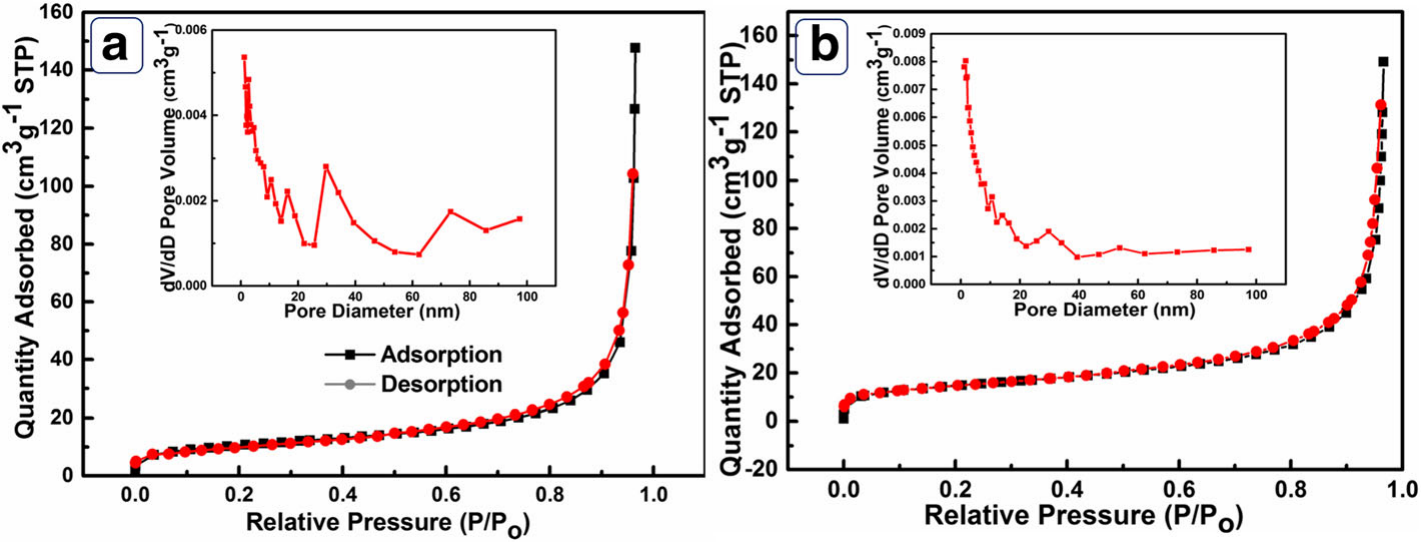
N_2_ adsorption-desorption isotherms of **a** Ni(OH)_2_ HPA and **b** Ni(OH)_2_ BHPA. Insets of **a** and **b** are the corresponding pore size distributions, respectively

The adsorption-desorption isotherm curve and the pore size distribution of Ni(OH)_2_ HPA and Ni(OH)_2_ BHPA are shown in Fig. 3. The SSA of Ni(OH)_2_ HPA is calculated to be 54.72 m^2^ g^−1^ based on the desorption curve, which is much larger than that of Ni(OH)_2_ BHPA (10.34 m^2^/g). The decrease of SSA can be attributed to the destruction of hollow structure and aggregation of the destroyed particles after ultrasonic treatment. The pore size distribution of Ni(OH)_2_ HPA and Ni(OH)_2_ BHPA both show regions below 10 nm, revealing the presence of nanopores between Ni(OH)_2_ nanoparticles. The pore size distribution of Ni(OH)_2_ HPA (inset of Fig. 3a) displays two concentrated regions of 20–40 nm and 60–85 nm, demonstrating the presence of micropores and mesopores. The micropores and mesopores might make the ion diffusion to active sites easier [21]. In the case of Ni(OH)_2_ BHPA (inset of Fig. 3b), a weak concentrated distribution is only investigated between 20 and 40 nm, indicating that the pore distribution of Ni(OH)_2_ BHPA is partly disordered. The decrease of SSA and destruction of ordered pore size may lead to difficulties in kinetics, resulting in poor electrocatalytic activity.

### Electrochemical Measurements

**Fig. 4:**
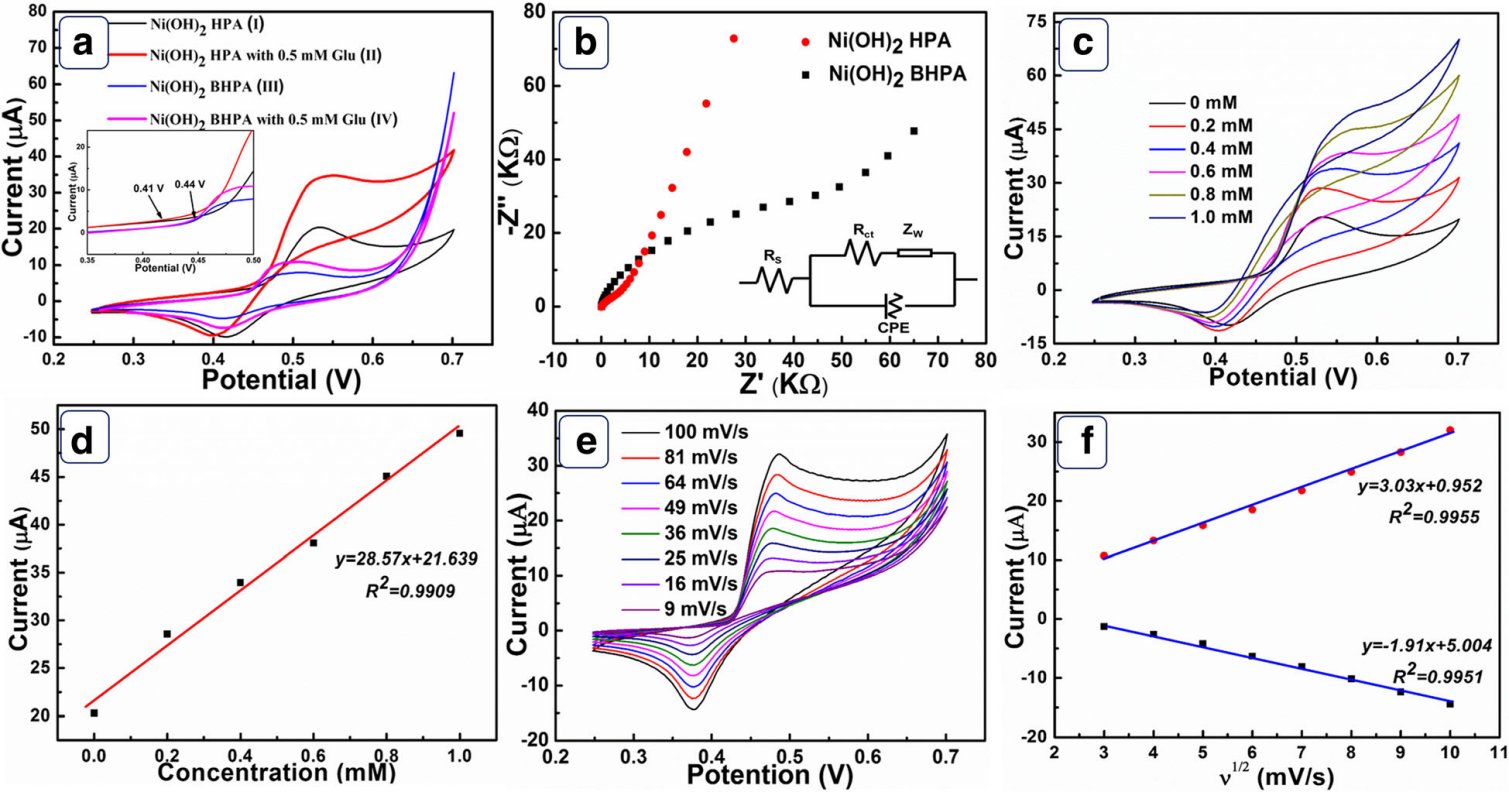
**a** CVs of Ni(OH)_2_ HPA (I, II) and Ni(OH)_2_ BHPA (III, IV) electrodes with (II, IV) and without (I, III) the presence of 0.5 mM glucose at 50 mV/s. **b** Nyquist diagrams EIS and equivalent circuit of Ni(OH)_2_ HPA and Ni(OH)_2_ BHPA. **c** CVs of Ni(OH)_2_ HPA electrode at various concentration of glucose and **d** the relationship between oxidation peak current and glucose concentration; **e** CVs of Ni(OH)_2_ HPA electrode at various scan rates with 0.5 mM glucose and **f** the relationship between peak current and square root of scan rates

**Scheme 1:**
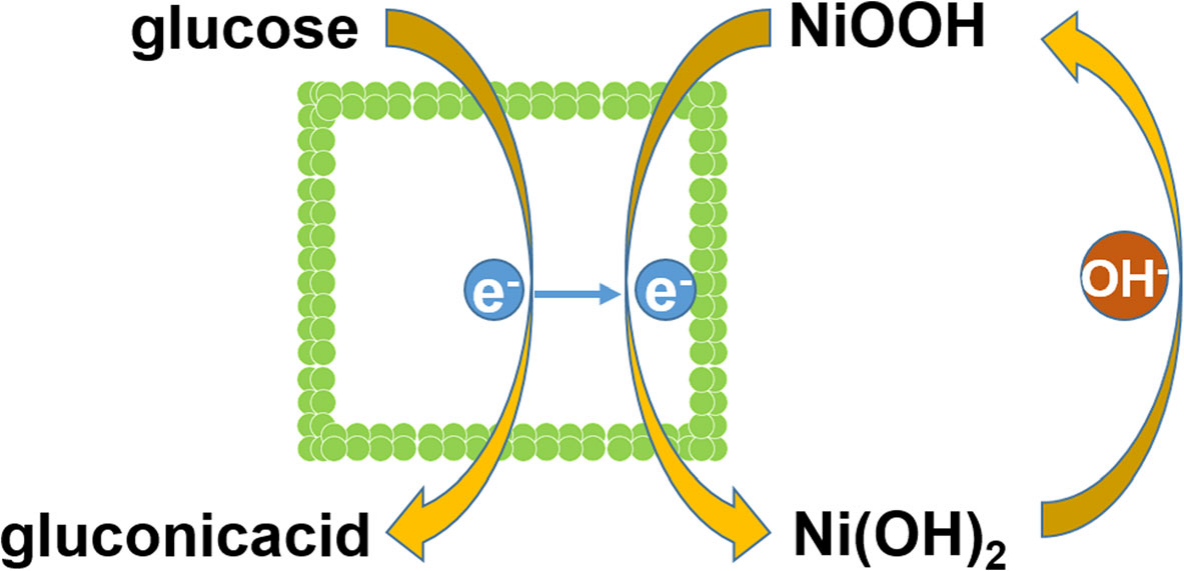
The schematic diagram of electrocatalytic mechanism

The electrocatalytic activity of Ni(OH)_2_ HPA and Ni(OH)_2_ BHPA was studied through the detection of glucose in 0.1 M NaOH. Figure 4a shows the CVs of Ni(OH)_2_ HPA and Ni(OH)_2_ BHPA electrodes with and without 0.5 mM glucose. Obviously, the redox peak current of Ni(OH)_2_ HPA (curve I) is higher than Ni(OH)_2_ BHPA (curve III) due to larger SSA. Upon addition of 0.5 mM glucose, the current responses of Ni(OH)_2_ HPA electrode (curve II) are higher than Ni(OH)_2_ BHPA electrode (curve IV). Otherwise, Ni(OH)_2_ HPA electrode shows lower onset potential (0.41 V) than that of Ni(OH)_2_ BHPA electrode (0.44 V). The higher electrocatalytic activity of Ni(OH)_2_ HPA can be attributed to high electron transfer rate, large SSA, and ordered pore structure provided by the hollow porous architecture. The electrocatalysis of glucose on Ni(OH)_2_ HPA electrode is driven by Ni(OH)_2_/NiOOH redox couple in alkaline medium on the basis of following reactions [22], and the corresponding schematic diagram is illustrated in Scheme 1.

4 $$ \mathrm{Ni}{\left(\mathrm{OH}\right)}_2+{\mathrm{OH}}^{-}\to \mathrm{Ni}\mathrm{OOH}+{\mathrm{H}}_2\mathrm{O}+{\mathrm{e}}^{-} $$

5 $$ \mathrm{Ni}\mathrm{OOH}+\mathrm{glucose}\to \mathrm{Ni}{\left(\mathrm{OH}\right)}_2+\mathrm{gluconicacid} $$

In order to confirm the kinetic advantages of hollow porous feature, EIS spectra of Ni(OH)_2_ HPA and Ni(OH)_2_ BHPA were measured (Fig. 4b). The EIS spectrum is characterized by a semicircle in high-frequency and an incline in low-frequency region. As shown in Additional file 1: Table S1, Ni(OH)_2_ HPA electrode exhibits smaller internal resistance (*Rs*) and electron transfer resistance (*Rct*) than Ni(OH)_2_ BHPA. Moreover, the Warburg impedance (*Zw*) of Ni(OH)_2_ HPA is larger than that of Ni(OH)_2_ BHPA, indicating more effective mass transfer rate. The difficulties in mass transfer kinetics of Ni(OH)_2_ BHPA can be ascribed to the destruction of ordered diffusion channels and aggregation of the broken cubes. In conclusion, Ni(OH)_2_ HPA electrode exhibits advantages in both electron and mass transfer kinetics compared to Ni(OH)_2_ BHPA. Figure 4c is the CVs of Ni(OH)_2_ HPA electrode in 0.1 M NaOH with different glucose concentration at 50 mV/s. The oxidation peak current linearly increases with the glucose concentration (Fig. 4d), revealing applications in electrochemical glucose sensors. The CVs of Ni(OH)_2_ HPA electrode with 0.5 mM glucose under different scan rates were recorded in Fig. 4e. As shown in Fig. 4f, the peak current linearly depends on the square root of scan rates, revealing diffusion controlled electrochemical process.

**Fig. 5:**
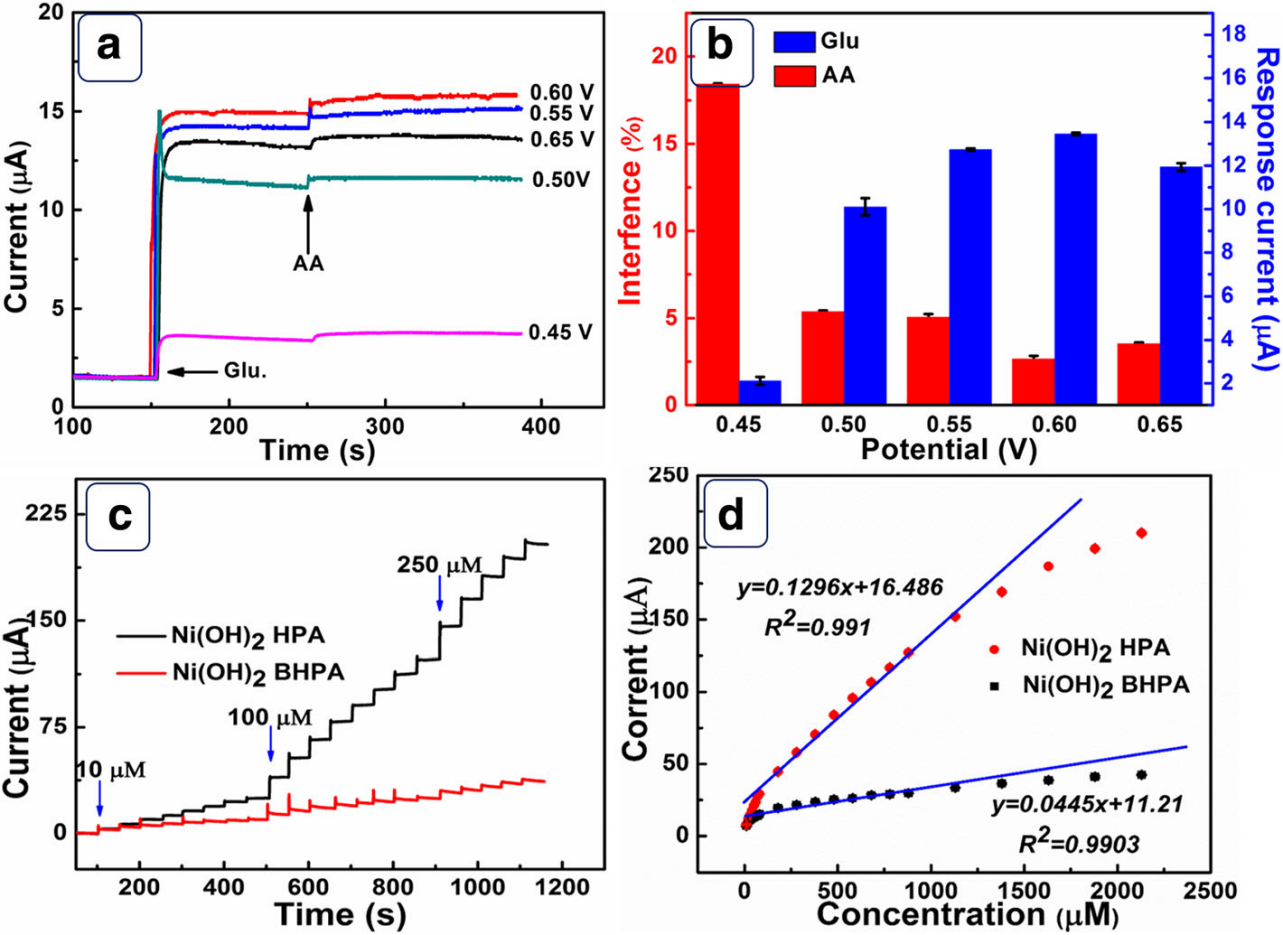
**a** Amperometric response of Ni(OH)_2_ HPA electrode at different potentials with the addition of 0.1 mM glucose and 0.01 mM AA. **b** The response current of glucose and AA at different potentials; **c** CA of Ni(OH)_2_ HPA and Ni(OH)_2_ BHPA electrodes at 0.6 V with the successive addition of glucose. **d** The relationship between response current and glucose concentration

In order to confirm the optimized working potential, the current response of glucose and the interference of AA were considered at different potentials (Fig. 5a). From the statistics data displayed in Fig. 5b, Ni(OH)_2_ HPA electrode exhibits minimum interference to AA and maximum current response to glucose at 0.6 V. Thus, 0.6 V was selected as the optimized working potential. Figure 5c shows the amperometric response curves of Ni(OH)_2_ HPA and Ni(OH)_2_ BHPA electrodes at 0.6 V. Ni(OH)_2_ HPA electrode presents more sensitive response to glucose than Ni(OH)_2_ BHPA electrode. Figure 5d is the corresponding calibration curves of Ni(OH)_2_ HPA and Ni(OH)_2_ BHPA electrodes. For Ni(OH)_2_ HPA electrode, the results show a good linear region between 0.08 mM and 1.13 mM. The fitting equation is *y =* 0.1296*x +* 16.486 (*R*_*2*_ *=* 0.991). By accurate calculation, Ni(OH)_2_ HPA electrode has a sensitivity of 1843 μA mM^−1^ cm^−2^, which is higher than Ni(OH)_2_ BHPA electrode (632 μA mM^−1^ cm^−2^). The detection limit of Ni(OH)_2_ HPA electrode is calculated to be 0.23 μM (S/*N* = 3), which is lower than Ni(OH)_2_ BHPA (0.67 μM). As displayed in Additional file 1: Figure S3, Ni(OH)_2_ HPA electrode presents a shorter response time (1.4 s) compared to Ni(OH)_2_ BHPA electrode (1.8 s). The analytical performances of Ni(OH)_2_ HPA electrode are compared with other Ni(OH)_2_-based electrodes, and the data are listed in Table 1. Notably, Ni(OH)_2_ HPA electrode presents higher electroactivity towards glucose in terms of high sensitivity, low detection limit, and rapid response, indicating great potential applications as an electrochemical glucose detection electrode.

**Table 1:** Comparison of researched electrode with reported nonenzymatic glucose sensors based on Ni(OH)_2_

Electrode	Sensitivity (μA mM^−1^ cm^−2^)	Detection limit (μM)	Response time (s)	Reference
Ni(OH)_2_ HPA/GCE	1843	0.23	1.4	This work
Ni(OH)_2_-CNT^a^-PVDF composite	–	23	3	[22]
Ni(OH)_2_/nanoflowers	265.3	0.5	3	[24]
Ni(OH)_2_/TiO_2_	192.5	8	1	[25]
Ni(OH)_2_ NP/MoS_*x*_	162	5.8	2	[26]
Nano Ni(OH)_2_ platelet-like	202	6	5–7	[27]
Ni(OH)_2_/ECF^b^	1342.2	0.1	3	[28]
Ni(OH)_2_-graphene	494	0.6	2	[29]
Ni(OH)_2_@oPPyNW^c^	1049.2	0.3	10	[30]
Ni(OH)_2_/Au/GCE	371.2	0.92	4	[31]

^a^Carbon nanotubes

^b^Electrospun carbon nanofiber

^c^Over-oxidized polypyrrole nanowires

**Fig. 6:**
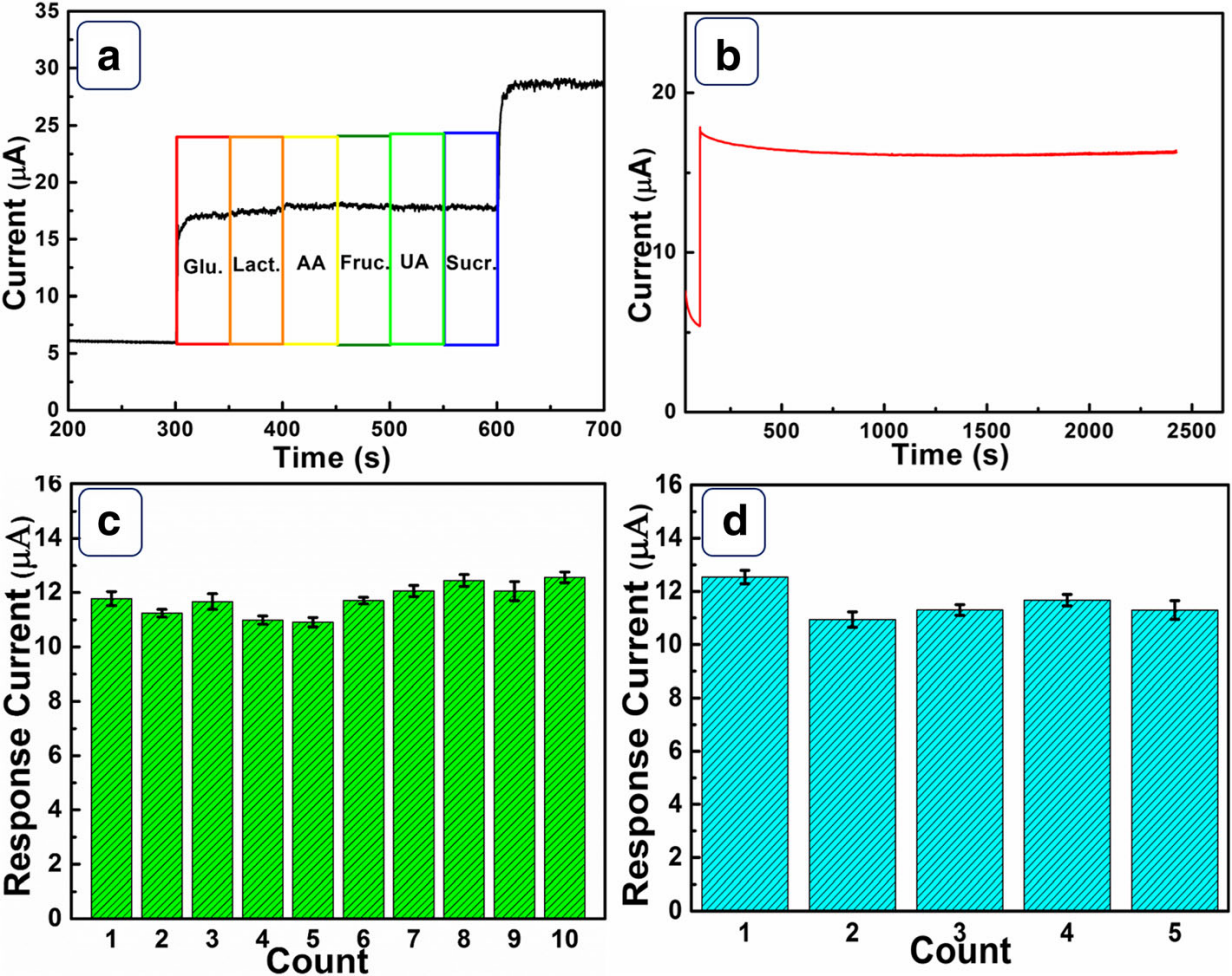
**a** The selectivity measurement of Ni(OH)_2_ HPA electrode at 0.6 V. The added glucose and all the interfering species are 0.1 mM and 0.01 mM, respectively. **b** The stability of Ni(OH)_2_ HPA electrode within 2400 s. **c** Ten measurements of one Ni(OH)_2_ HPA electrode towards 0.1 mM glucose. **d** Current responses of five Ni(OH)_2_ HPA electrodes towards 0.1 mM glucose

Common interferents in human blood, including Lact., Suct., Fruct., UA, and AA, are involved to evaluate the selectivity of Ni(OH)_2_ HPA electrode [23]. As displayed in Fig. 6a, no more than 3.8% interferences are observed for all the interferents. The second current response for glucose retains 98.1% of its first signal. Figure 6b displays the amperometric response of Ni(OH)_2_ HPA electrode towards 0.1 mM glucose within 2400 s at 0.60 V. The final response signal still retains approximately 93.5% of its original data, revealing an excellent long-term stability of Ni(OH)_2_ HPA electrode. In Fig. 6c, current responses for one Ni(OH)_2_ HPA electrode were tested for ten times. The signals display a relatively standard deviation (RSD) of 4.8%, demonstrating outstanding reproducibility. Moreover, the five Ni(OH)_2_ HPA electrodes exhibit a satisfying RSD of 5.3% (Fig. 6d). Ni(OH)_2_ HPA electrode possesses excellent selectivity, satisfying stability and reproducibility, demonstrating attractive applications in electrochemical glucose sensors.

## Conclusions

We have used a facile strategy inspired by the CEP principle to controllably fabricate uniform Ni(OH)_2_ HPA at room temperature. Ni(OH)_2_ HPA presents large SSA, ordered diffusion channels, and high structure stability. As a electrochemical detection electrode for glucose, Ni(OH)_2_ HPA exhibits higher sensitivity of 1843 μA mM^−1^ cm^−2^, faster response time (1.4 s), and lower detection limit of 0.23 μM compared to broken sample (1.8 s, 0.67 μM). The Ni(OH)_2_ HPA electrode presents improved electrochemical sensing performance towards glucose, revealing promising feature for the practical analytical application. The hollow porous architecture is also confirmed as an effective strategy to obtain high-performance electrocatalysts.

## Additional file

Additional file 1: **Figure S1.** (a) SEM and (b) XRD pattern of the prepared Cu_2_O. Figure S2 The SEM image of Ni(OH)_2_ BHPA. Figure S3 The detection limit of Ni(OH)_2_ HPA and Ni(OH)_2_ BHPA. Table S1 Comparison of researched Ni(OH)_2_ HPA electrode with Ni(OH)_2_ BHPA about EIS. (DOCX 1575 kb)

## Abbreviations

AA: l-ascorbic acid
BET: Brunauer-Emmett-Teller
CA: Chronoamperometry
CEP: Coordinating etching and precipitating
CNT: Carbon nanotubes
ECF: Electrospun carbon nanofiber
EIS: Electrochemical impedance spectroscopy
FESEM: Field emission scanning electron microscope
Fruc.: Fructose
GCE: Glassy carbon electrode
Glu.: Glucose
HPA: Hollow porous architecture
Lact.: Lactose
Ni(OH)_2_ BHPA: Broken Ni(OH)_2_ HPA
oPPyNW: Over-oxidized polypyrrole nanowires
PVP: Polyvinyl pyrrolidone
*Rct*: Electron transfer resistance
*Rs*: Internal resistance
RSD: Relatively standard deviation
SAED: Selected area electron diffraction
SSA: Specific surface area
Sucr.: Sucrose
UA: Uric acid
XPS: X-ray photoelectron spectrometer
XRD: X-ray diffraction
*Zw*: Warburg impedance
