# Supplementary material for: Rational Design of Ni(OH)2 Hollow Porous Architecture for High-Sensitivity Enzyme-Free Glucose Sensor
Source: Nanoscale Res Lett. 2018 Oct 29;13:342. doi: 10.1186/s11671-018-2726-8 (PMC6206306; doi:10.1186/s11671-018-2726-8)
Supplement: Supplementary file 1 — Figure S1. (a) SEM and (b) XRD pattern of the prepared Cu2O. Figure S2 The SEM image of Ni(OH)2 BHPA. Figure S3 The detection limit of Ni(OH)2 HPA and Ni(OH)2 BHPA. Table S1 Comparison of researched Ni(OH)2 HPA electrode with Ni(OH)2 BHPA about EIS. (DOCX 1575 kb) [file 11671_2018_2726_MOESM1_ESM.docx]

**Rationally design of Ni(OH)_2_ hollow porous architecture for high sensitive enzyme-free glucose sensor**

Liangliang Tian^1^, Gege He^1,2^, Meijing Chen^1*^, Jinbiao Wang^1*^, Yucen Yao^1^, Xue Bai^1^

^1^ Research Institute for New Materials Technology, Chongqing University of Arts and Sciences, Chongqing, People's Republic of China

^2^ School of Science, MOE Key Laboratory for Non-equilibrium Synthesis and Modulation of Condensed Matter, State Key Laboratory for Mechanical Behavior of Materials, Xi'an Jiaotong University, Xi'an, People's Republic of China

**^*^Corresponding author:**

**Meijing Chen**

Email: cmjftxlj@163.com

**Jinbiao Wang**

Email: wlsf108@163.com


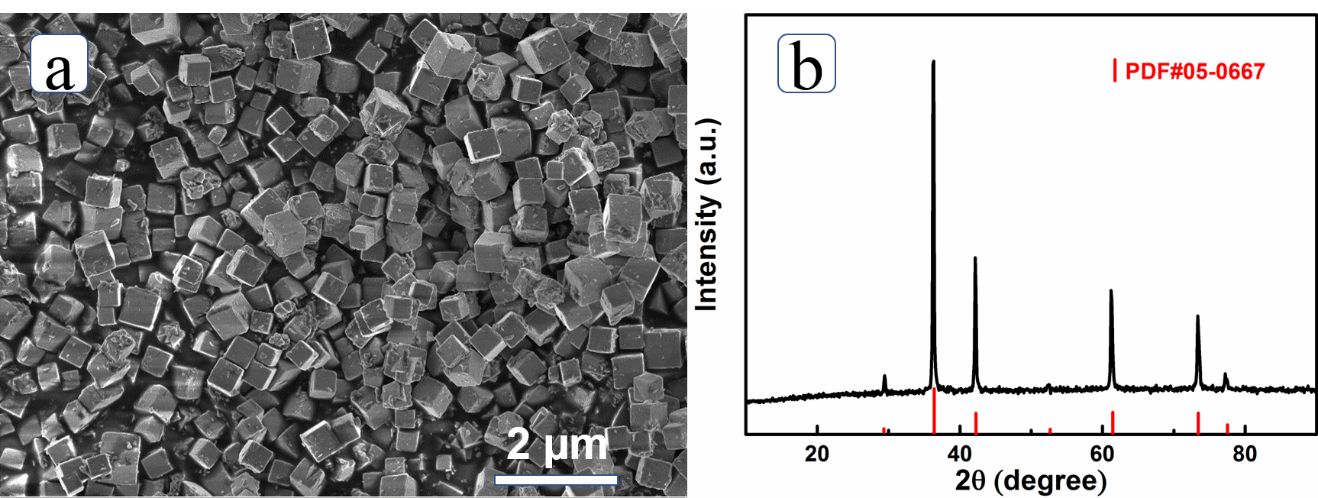


Figure S1. (a) SEM and (b) XRD pattern of the prepared Cu_2_O.


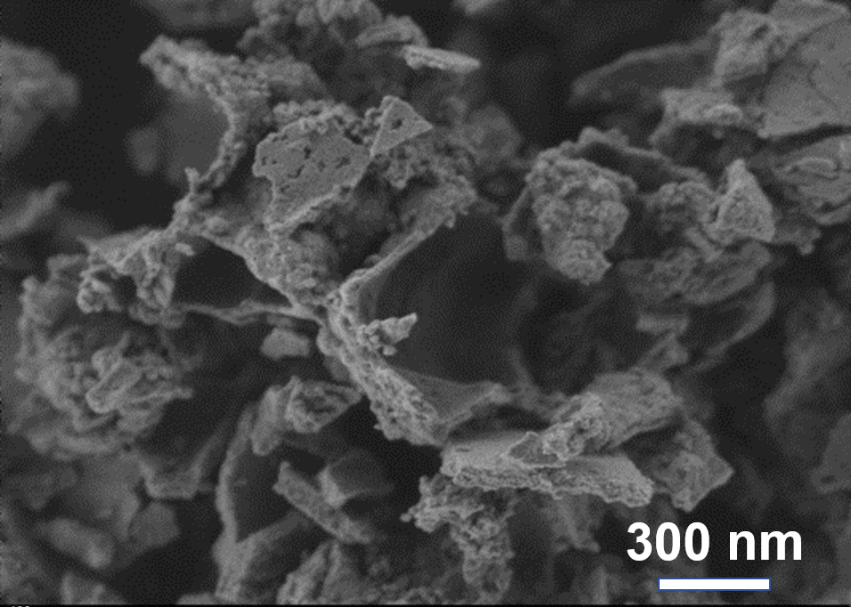


Figure S2. The SEM image of Ni(OH)_2_ BHPA.





Figure S3. The detection limit of Ni(OH)_2_ HPA and Ni(OH)_2_ BHPA.

Table S1. Comparison of researched Ni(OH)_2_ HPA electrode with Ni(OH)_2_ BHPA about EIS.

| Samples | Ni(OH)_2_ BHPA | Ni(OH)_2_ HPA |
| --- | --- | --- |
| *R_s_* | 144 Ω | 66 Ω |
| *R_ct_* | 99.6 KΩ | 33.9 KΩ |
| *Z_w_* | 10.4 µMh_o_ (N=0.89) | 61.3 µMh_o_ (N=0.896) |

*The sample of EIS data estimated error less than 5%.
